# Differential controls on soil carbon density and mineralization among contrasting forest types in a temperate forest ecosystem

**DOI:** 10.1038/srep22411

**Published:** 2016-03-01

**Authors:** Ye-Ming You, Juan Wang, Xiao-Lu Sun, Zuo-Xin Tang, Zhi-Yong Zhou, Osbert Jianxin Sun

**Affiliations:** 1 College of Forest Science, Beijing Forestry University, Beijing 100083, China; 2 Institute of Forestry and Climate Change Research, Beijing Forestry University, Beijing 100083, China; 3 College of Forestry, Guangxi University, Nanning, Guangxi 530004, China

## Abstract

Understanding the controls on soil carbon dynamics is crucial for modeling responses of ecosystem carbon balance to global change, yet few studies provide explicit knowledge on the direct and indirect effects of forest stands on soil carbon via microbial processes. We investigated tree species, soil, and site factors in relation to soil carbon density and mineralization in a temperate forest of central China. We found that soil microbial biomass and community structure, extracellular enzyme activities, and most of the site factors studied varied significantly across contrasting forest types, and that the associations between activities of soil extracellular enzymes and microbial community structure appeared to be weak and inconsistent across forest types, implicating complex mechanisms in the microbial regulation of soil carbon metabolism in relation to tree species. Overall, variations in soil carbon density and mineralization are predominantly accounted for by shared effects of tree species, soil, microclimate, and microbial traits rather than the individual effects of the four categories of factors. Our findings point to differential controls on soil carbon density and mineralization among contrasting forest types and highlight the challenge to incorporate microbial processes for constraining soil carbon dynamics in global carbon cycle models.

Quantification of changes in soil carbon storage in space and time has proven to be a challenging issue in terrestrial ecosystem budgeting as well as in global carbon cycle research. In this regard model simulations based on ecosystem processes provide a feasible and cost-effective tool for large scale mapping of soil carbon stocks and dynamics. However, the adequacy in model representation of the complex dynamics of soil carbon processes depends on explicit knowledge on mechanistic constraints of soil carbon transformation and turnover by various biotic and environmental factors^1^. Soil carbon storage is a consequence of plant litter and other organic detritus inputs and controls of organic matter decomposition by a series of biotic, chemical and physical factors^2^,^3^. With increasing data availability and advancement in field manipulation studies, it has become better understood that direct biological, physical and chemical processes play prominent roles in regulating soil carbon processes over short time periods and at local scales^4^,^5^,^6^, and that long-term trends and spatial variations of soil carbon storage likely reflect the impacts of changes in biological, environmental and social factors^7^.

Mechanisms underlying the directional changes in soil carbon toward accumulation and stabilization have recently emerged as a focal point of interest in soil carbon research in the context of mitigating greenhouse gas emissions in natural ecosystems^8^. Climatic factors are known to dominate in the environmental effects on soil carbon storage and turnover across forests of varying biogeoclimatic zones^9^,^10^; temperature and water are recognized as two critical determinants of soil carbon cycling through alteration of soil physicochemical properties^2^,^11^ and controls on microbial community structure and activities^1^,^12^,^13^,^14^. Other biological and biophysical factors, such as plant traits, soil biota, substrate quality and stand structure, may prevail as dominant drivers of soil carbon dynamics at a local scale^14^,^15^,^16^. Although numerous studies have been conducted to determine the effects of various biotic and environmental factors on soil carbon processes^17^,^18^,^19^,^20^, there lacks an explicit understanding on the underlying mechanisms of multiple constraints on, and regulations of, soil carbon dynamics. Differentiating the specific effects of biotic and environmental factors on soil carbon transformation and turnover has posed a critical challenge because of the complex interplays among various biotic and environmental factors in shaping the ecosystem processes and lack of a clear understanding on the feedbacks between the biological and physiochemical processes^7^.

There is growing evidence that forest type and stand developmental stage can impose marked effects on soil carbon cycling as results of differential plant traits and varying canopy structures as well as interactions with soil processes^11^,^15^,^19^, but it is unclear how factors associated with stand characteristics would interact with soil biota to shape the soil carbon dynamics across contrasting forest types and varying stand developmental stages. Variations in litter chemistry and quality are widely recognized to explain species-dependent effects on soil carbon cycling^16^,^21^; whereas stand structure may affect forest floor microclimate, thereby influencing soil carbon dynamics via environmental impacts on soil microbial processes. Differences in plant traits may also influence soil carbon cycling by determining the structure and activty of soil biota, especially by facilitating varying assemblages of bacterial and fungal communities^14^,^18^. Previous studies have established that soil respiration, the rate of soil carbon accumulation and soil microbial activity could all vary with ecosystem succession or stand dynamics^15^,^22^,^23^,^24^,^25^. However, research to date has rarely linked the soil microbial community structure with functioning in explaining tree species mediated variations in soil carbon dynamics.

Soil microbial community composition and activities are found to vary across different biomes conditioned by the regional climate^12^,^13^, and significant differences in the patterns of soil carbon dynamics have been reported to occur between broadleaved and coniferous forests that differ markedly in the physical and chemical properties of litter^26^,^27^. However, the effects of forest type and stand developmental stage on belowground processes relating to soil carbon dynamics vary among different studies. For example, while some studies show that the patterns of soil carbon cycling vary with forest types and stand developmental stages^22^,^23^,^24^, there are findings that forest stand age is not strongly associated with microbial activities driving the soil carbon processes^22^,^28^. The mechanisms underlying the effects of forest type and stand developmental stage on soil carbon cycling may involve differential biological traits and environmental factors as well as plant-site interactions^29^. To date it is unclear whether the influences of plant traits on soil carbon are overwhelmed by that of environmental factors, and the differential roles of plant traits and environmental factors in regulating soil carbon cycling are still poorly elucidated. In many cases, our knowledge on the factors driving soil carbon transformation and turnover remains more qualitative than quantitative, and it is difficult to parameterize ecosystem process models concerning soil carbon dynamics^3^.

This study aims to quantify the specific effects of biotic (i.e. tree species and microorganisms) and abiotic (i.e. microclimate and soil) factors on soil carbon processes as affected by forest type and stand developmental stage under a temperate climate. Investigations of variables relating to soil carbon processes and associated biotic and environmental factors were made across natural forest stands dominated by three different oak species, including *Quercus aliena* var. *acuteserrata* Maxim., *Quercus glandulifera* var. *brevipetiolata* Nakai., and *Quercus variabilis* Blume., and of a mixed pine/oak forest dominated by *Pinus armandii* Franch and *Q. aliena* var. *acuteserrata*, as well as in an age sequence of *Q. aliena* var. *acuteserrata* forests (i.e. ~40, ~80, and >160 yrs), in a temperate forest ecosystem in central China. Data were collected on a total of 27 variables for assessing the effects of biotic and edaphic factors on soil carbon density and mineralization, categorized into (1) microclimate (MC): consisting of soil temperature at 10 cm depth (T_soil10_), soil water content (%SWC), and average slope of each plot (%Slope) and stem basal area (BA) for capturing potential influences of topography and stand structure; (2) soil factor (S): consisting of soil total nitrogen (TN_soil_), ammonium nitrogen (NH_4_-N), nitrate nitrogen (NO_3_-N), dissolved organic carbon (DOC), soil carbon to nitrogen ratio (C/N_soil_), soil pH, and particle distributions of sand (%Sand), silt (%Silt) and clay (%Clay); (3) tree species traits (T): consisting of fine root biomass (FR), forest floor litter mass (Litter_mass_), annual litterfall (Litter_fall_), litter lignin content (%lignin_litter_), root lignin content (%lignin_root_), litter carbon content (%C_litter_), litter nitrogen content (%N_litter_), litter carbon to nitrogen ratio (C/N_litter_), root carbon to nitrogen ratio (C/N_root_), litter lignin to nitrogen ratio (lignin/N_litter_), and root lignin to nitrogen ratio (lignin/N_root_); and (3) soil microbial traits (M): consisting of microbial biomass carbon (MBC), microbial biomass nitrogen (MBN), and microbial carbon to nitrogen ratio (C/N_mic_). We also measured microbial respiration (MR) and activities of four soil extracellular enzymes involved in degrading cellulose (β-1,4-glucosidase [BG]), chitin (β-1,4-N-acetylglucosaminidase [NAG]), and lignin (phenol oxidase [PO] and peroxidase [PER]).

We anticipated that variations in soil carbon dynamics among forest types and across stand developmental stages likely reflect direct effects by the quality and quantity of plant-derived soil organic carbon, and that tree-soil feedbacks would impose independent and secondary effects through modification of forest floor microclimate and soil microbial communities. The specific objectives of our study were to determine how the soil microbial communities and soil carbon dynamics would vary with changes in forest type and stand developmental stage under temperate climate, and to determine the specific effects of biotic and environmental factors on soil carbon dynamics associated with different types of forest stands.

## Results

**Figure 1:**
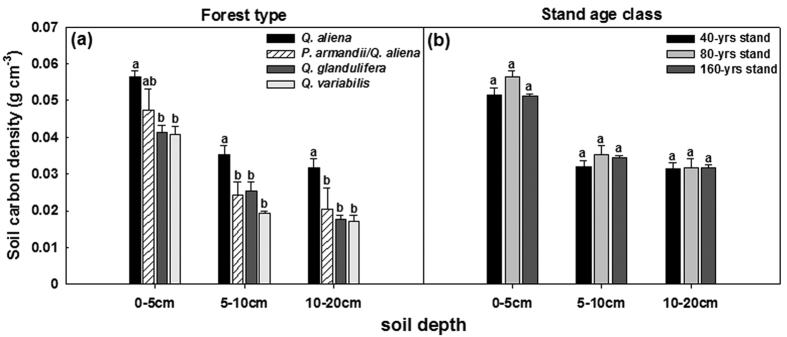
Soil carbon density in 0–5, 5–10 and 10–20 cm soil layers in plots of contrasting forest types and stand age classes. Error bars represent 1 SE (n = 3). Different letters above the error bars indicate significant differences among forest types/stand age classes within given soil layers (*P* < 0.05).

**Figure 2:**
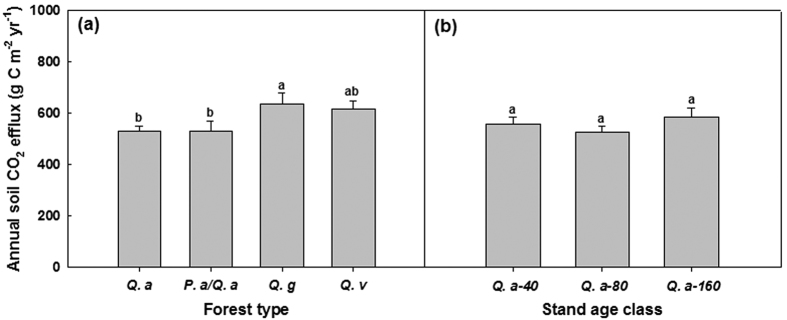
Soil CO_2_ efflux in plots of contrasting forest types and stand age classes. Error bars represent 1 SE (n = 3). Different letters above the error bars indicate significant differences among forest types/stand age classes (*P* < 0.05). *Q.a*: *Q. aliena* forest; *P.a/Q.a*: mixed *P. armandii*/*Q. aliena* forest; *Q.g*: *Q. glandulifera* forest; *Q.v*: *Q. variabilis* forest; *Q.a*-40: 40-year *Q. aliena* forest; *Q.a*-80: 80-year *Q. aliena* forest; *Q.a*-160: >160-year *Q. aliena* forest.

Significant differences in soil carbon density were found among forest types in each of the three soil layers investigated (*P* < 0.05; d.f. = 3, 8); the values were mostly greatest in the *Q. aliena* plots and smallest in the *Q. variabilis* plots (Fig. 1a). Significant differences in annual soil respiration also occurred among forest types (*P* < 0.05; d.f. = 3, 8); the annual soil CO_2_ efflux was highest in the *Q. glandulifera* plots, followed by the *Q. variabilis* plots, and was similar between *Q. aliena* and *P. armandii/Q. aliena* plots (Fig. 2a). No significant difference was found among the stand age classes of *Q. aliena* forest in either soil carbon density (Fig. 1b) or annual soil respiration (Fig. 2b).

**Figure 3:**
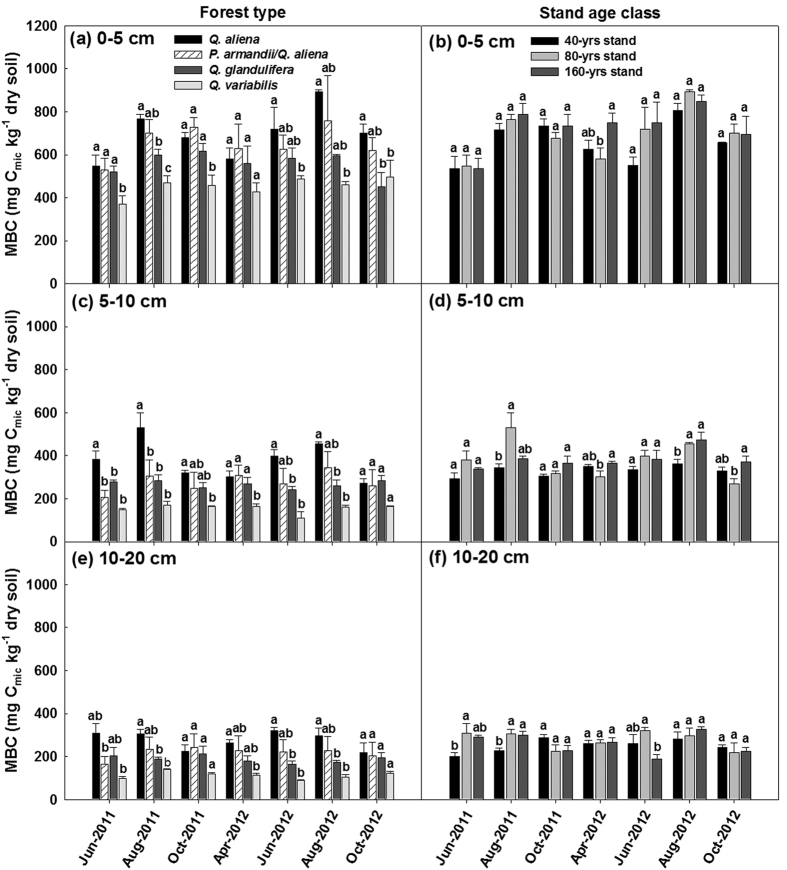
Soil microbial biomass C (MBC) in 0–5, 5–10, 10–20 cm soil layers in plots of contrasting forest types and stand age classes during the growing seasons in 2011 and 2012. Error bars represent 1 SE (n = 3). Different letters above the error bars indicate significant differences among forest types/stand age classes for each sampling time (*P* < 0.05).

**Figure 4:**
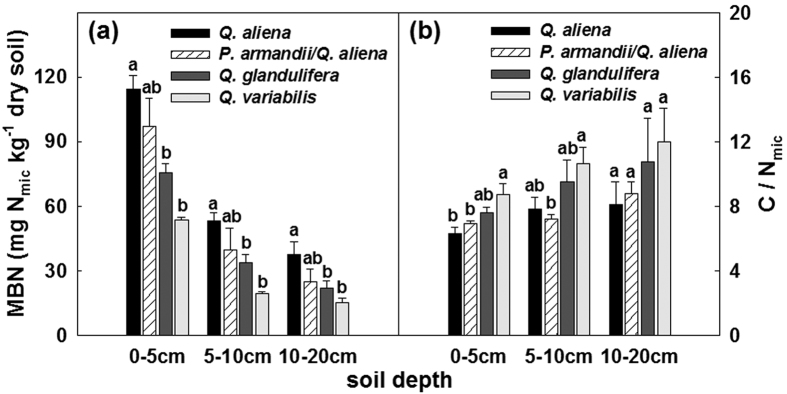
Mean values of microbial biomass N (MBN) and microbial C to N ratio (C/N_mic_) in 0–5, 5–10, 10–20 cm soil layers in plots of contrasting forest types during the growing seasons from 2011 to 2012. Error bars represent 1 SE (n = 3). Different letters above the error bars indicate significant differences among forest types within given soil layers (*P* < 0.05).

**Figure 5:**
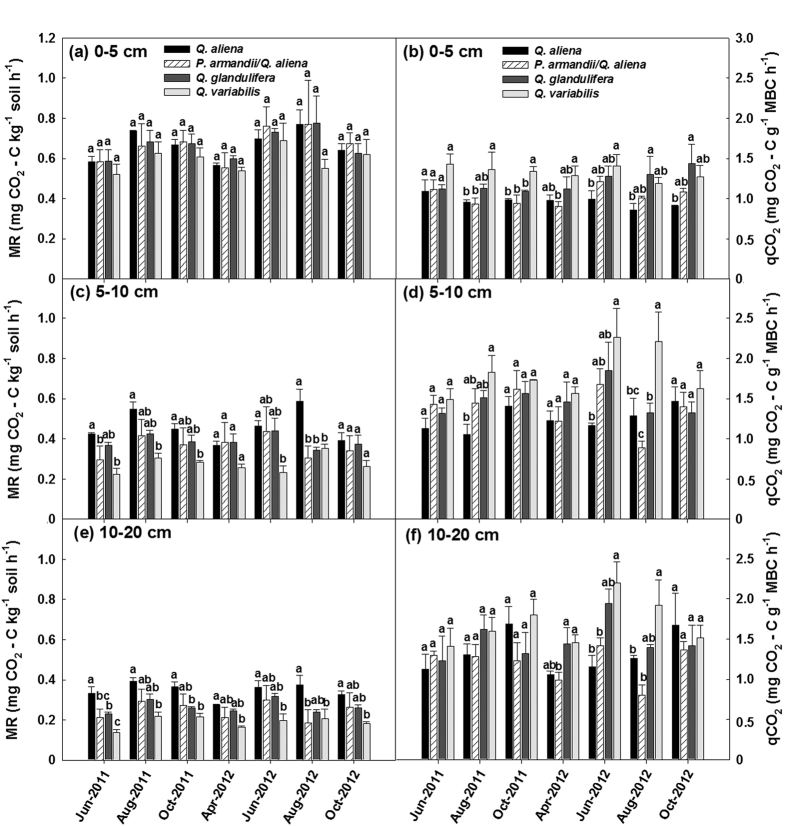
Soil microbial respiration (MR) and microbial metabolic quotient (qCO_2_) in the 0–5, 5–10 and 10–20 cm soil layers in plots of contrasting forest types during the growing seasons in 2011 and 2012. Error bars represent 1 SE (n = 3). Different letters above the error bars indicate significant differences among forest types for each sampling time (*P* < 0.05).

The four forest types significantly differed (*P* < 0.05; d.f. = 3, 8; Table S1) in distributions of sand and clay and NO_3_-N in all three soil layers except %Clay in the 0–5 cm soil layer and NO_3_-N in the 5–10 cm soil layer (Table S1), and highly significantly differed (*P* < 0.01 or 0.001; d.f. = 3, 8; Table S1) in TN_soil_, soil pH and C/N_soil_. Only the values of TN_soil_ were found to significantly differ (*P* < 0.05; d.f. = 2, 6; Table S1) among the three stand age classes of the *Q. aliena* stands. The *Q. aliena* and *P. armandii*/*Q. aliena* plots had higher values of %SWC, TN_soil_, NO_3_-N, and pH, but lower values of T_soil10_ and C/N_soil_, than *Q. glandulifera* and *Q. variabilis* plots (Tables S2 and S3). Overall no significant differences were found among stand age classes of the *Q. aliena* forest (Table S4 and S5). Results of repeated measures ANVOAs showed that the effect of forest type significantly varied with time of sampling on MBC (Greenhouse-Geisser corrected: *P* < 0.05; d.f. = 7.92, 21.15) and qCO_2_ (*P* < 0.05; d.f. = 18, 48) in the 5–10 cm soil layer, and on MR in the 5–10 cm soil layer (Greenhouse-Geisser corrected: *P* < 0.05; d.f. = 6.03, 16.05) and the 10–20 cm soil layer (Greenhouse-Geisser corrected: *P* < 0.05; d.f. = 4.86, 12.97) (Table S6). The *Q. aliena* and *P. armandii*/*Q. aliena* plots generally had higher values of MBC (Fig. 3a,c,e) and MBN (Fig. 4a) and lower values of C/N_mic_ (Fig. 4b) than both *Q. glandulifera* and *Q. variabilis* plots. The four forest types had similar rates of MR for soils of the top layer (0–5 cm depth), but differed in MR in the 5–10 cm and 10–20 cm soil layers (Fig. 5a,c,e). The values of qCO_2_ were mostly highest in the *Q. variabilis* plots, followed by the *Q. glandulifera* plots, and were mostly similar between the *Q. aliena* and *P. armandii*/*Q. aliena* plots (Fig. 5b,d,f). The three stand age classes of *Q. aliena* forest only significantly differed in MBC in the 5–10 cm soil layer (*P* < 0.05; d.f. = 2, 6; Table S6), and there was a tendency of increasing MBC and MR with stand development (Figs 3b,d,f and S2). However, the differences in microbial variables were not significant among stand age classes in any of the individual soil layers except MBC in the 5–10 cm soil layer (Figs S1 and S2).

**Figure 6:**
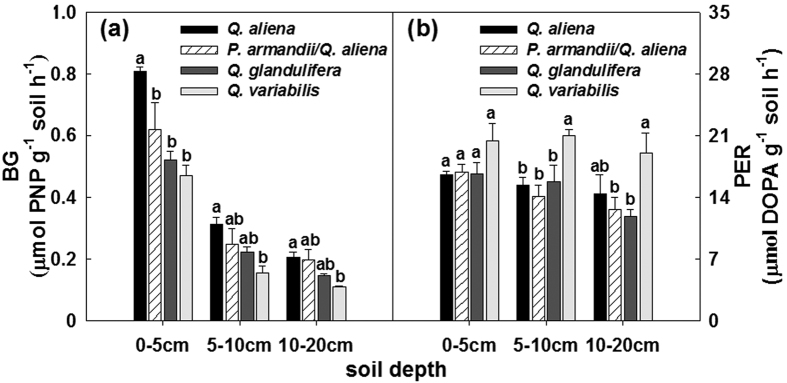
Mean values of activities of β-1,4-glucosidase (BG), and peroxidase (PER) in the 0–5, 5–10, 10–20 cm soil layers in plots of contrasting forest types during the growing seasons from 2011 to 2012. Error bars represent 1 SE (n = 3). Different letters above the error bars indicate significant differences among forest types within given soil layers (*P* < 0.05).

**Figure 7:**
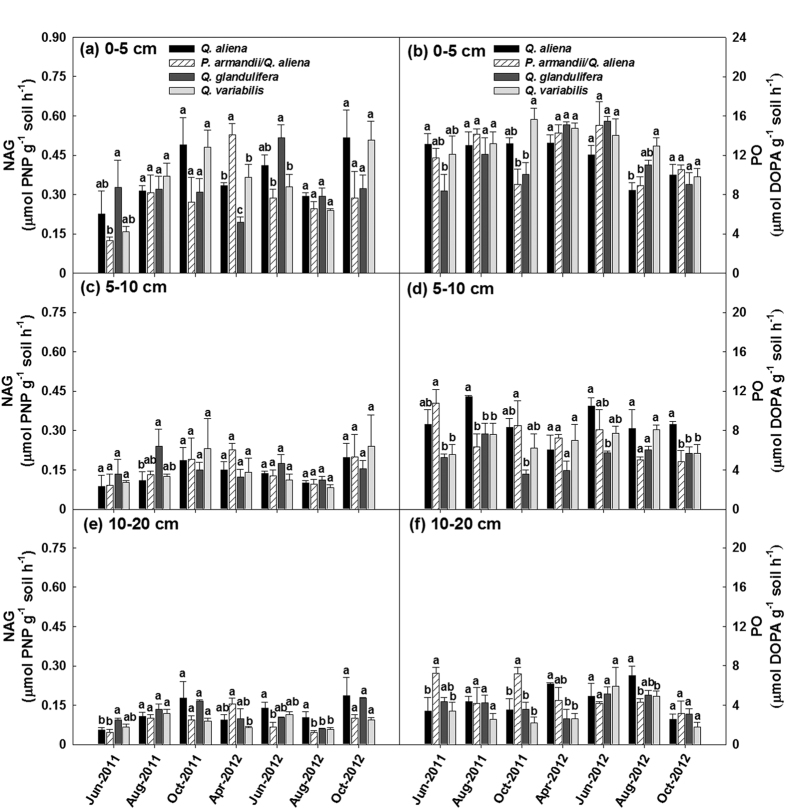
Activities of β-1,4-N-acetylglucosaminidase (NAG) and phenol oxidase (PO) in the 0–5, 5–10, 10–20 cm soil layers in plots of contrasting forest types during the growing seasons in 2011 and 2012. Error bars represent 1 SE (n = 3). Different letters above the error bars indicate significant differences among forest types for each sampling time (*P* < 0.05).

The total activities of BG, NAG, PO and PER were significantly affected by forest type within given soil layers, except PO and PER in the 0–5 cm soil layer and NAG in the 5–10 cm soil layer (*P* < 0.05; d.f. = 3, 8; Table S7). The BG activity was highest in the *Q. aliena* plots and lowest in the *Q. variabilis* plots (Fig. 6a); whereas the PER activity was higher in the *Q. variabilis* plots than in plots for other three forest types (Fig. 6b).The NAG and PO activities did not exhibit consistent variations among the forest types in any of the soil layers (Fig. 7); the effect of forest type significantly varied with time of sampling on NAG activity in the 0–5 cm soil layer (Greenhouse-Geisser corrected: *P* < 0.05; d.f. = 6.90, 18.43), and on PO activity in the 5–10 cm soil layer (Greenhouse-Geisser corrected: *P* < 0.05; d.f. = 8.28, 22.07) and the 10–20 cm soil layer (Greenhouse-Geisser corrected: *P* < 0.05; d.f. = 8.19, 21.84) (Table S7). Stand age class only affected the PER activity in the 5–10 cm soil layer (*P* < 0.05; d.f. = 2, 6; Table S7); there was an increasing BG activity with stand development (Fig. S3).

**Figure 8:**
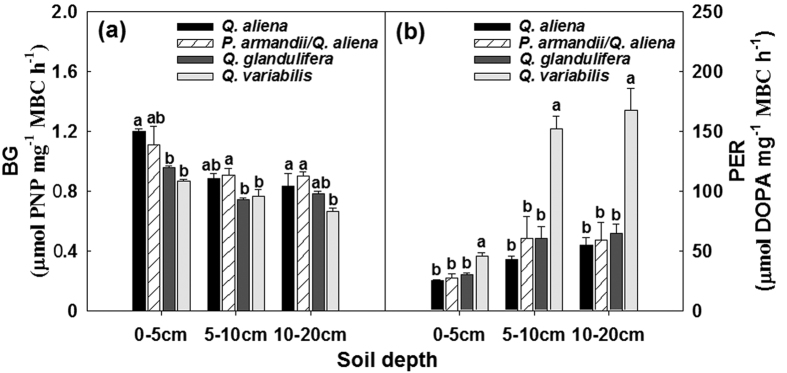
Mean values of specific activities of β-1,4-glucosidase (BG) and peroxidase (PER) in the 0–5, 5–10, 10–20 cm soil layers in plots of contrasting forest types during the growing seasons from 2011 to 2012. Error bars represent 1 SE (n = 3). Different letters above the error bars indicate significant differences among forest types within given soil layers (*P* < 0.05).

**Figure 9:**
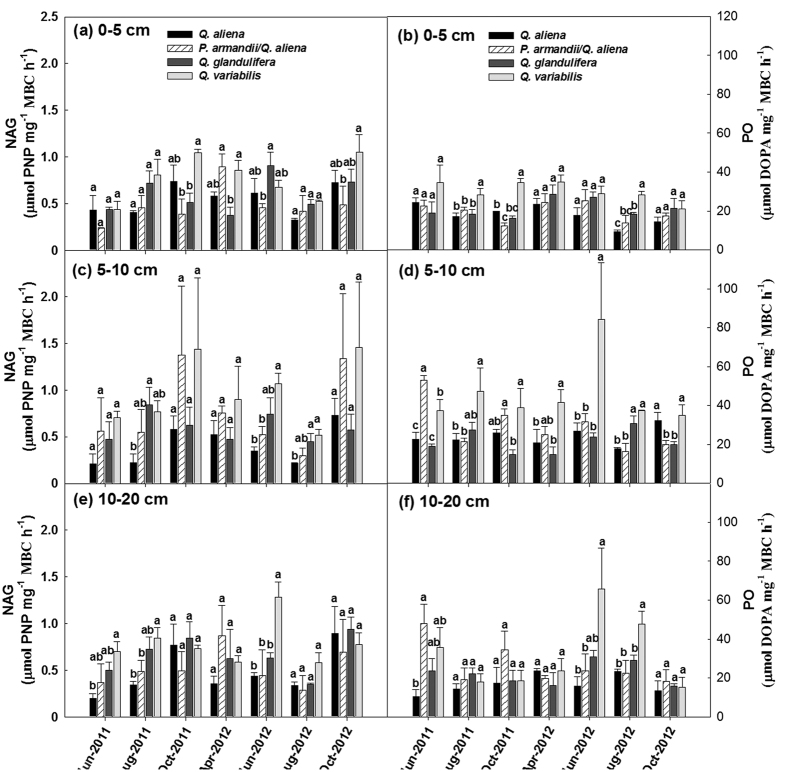
Specific activities of β-1,4-N-acetylglucosaminidase (NAG) and phenol oxidase (PO) in the 0–5, 5–10, 10–20 cm soil layers in plots of contrasting forest types during the growing seasons in 2011 and 2012. Error bars represent 1 SE (n = 3). Different letters above the error bars indicate significant differences among forest types for each sampling time (*P* < 0.05).

The microbial biomass-specific enzyme activities varied significantly among forest types in each of the three soil layers investigated, except NAG in the 5–10 cm soil layer and 10–20 cm soil layer (*P* < 0.05; d.f. = 3, 8), but not across stand age classes (Table S8). The *Q. aliena* and *P. armandii*/*Q. aliena* plots generally had higher microbial biomass-specific BG activity (Fig. 8a), whereas the *Q. glandulifera* and *Q. variabilis* plots were characterized by relatively higher microbial biomass-specific activity of PER (Fig. 8b). The effect of forest type significantly varied with time of sampling on microbial biomass-specific NAG activity in the 0–5 cm soil layer (*P* < 0.05; d.f. = 18, 48) and the 10–20 cm soil layer (*P* < 0.05; d.f. = 18, 48), and on the microbial biomass-specific PO activity in the 10–20 cm soil layer (Greenhouse-Geisser corrected: *P* < 0.05; d.f. = 7.08, 18.89) (Table S8; Fig. 9).

**Figure 10:**
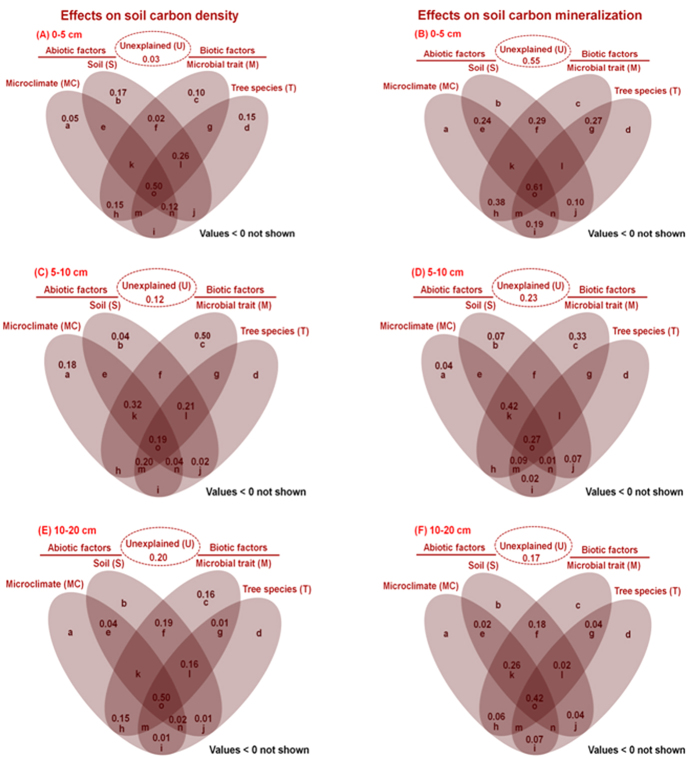
Individual and shared effects of microclimatic, edaphic, microbial traits and tree species factors on (A) soil carbon density and (B) soil carbon mineralization as derived from variation partitioning analysis. Values are proportions of variations explainable by individual (letters a through d) and shared (letters e through o) effects of the four categories of factors. The adjusted *R*^*2*^ values < 0 were not shown and were interpreted as a zero value.

The variation partitioning analysis revealed that the four categories of factors, i.e. microclimate (MC), soil (S), microbial traits (M), and tree species (T), collectively explained a high proportion of variations in soil carbon density (Fig. 10A,C,E); about half of the variations in soil carbon density were explainable by the shared effects of the four categories of factors in the upper soil layer (0–5 cm; Fig. 10A, fraction o)and lower soil layer (10–20 cm; Fig. 10E, fraction o), whereas only about 19% of the variations in soil carbon density were explainable by the shared effect of the four categories of factors in the middle soil layer (5–10 cm; Fig. 10C, fraction o). The effects of individual categories of factors on soil carbon density were most apparent in the upper 0–5 cm soil layer (Fig. 10A, Fraction b, c and d), but were negligible in the middle and lower soil layers, except the M factor (50% and 16% explained, respectively; Fig. 10C,E, Fraction c) and the MC in the middle soil layer (Fig. 10C, Fraction a). The shared effect of MC, S and M factors explained a large proportion of the variations in soil carbon density in the 5–10 cm soil layer (Fig. 10C, fraction k), and the shared effect of S, M and T factors explained 21% of the variations in soil carbon density in the 5–10 cm soil layer (Fig. 10C, fraction l). The shared effect of MC and M factors explained a large proportion (about 15%) of the variations in soil carbon density in the 0–5 cm and 10–20 cm soil layers (Fig. 10A,E, fraction h), and the shared effect of S and M factors also explained a large proportion of the variations in soil carbon density in the 10–20 cm soil layer (Fig. 10E, fraction f).The shared effects of other combinations of factors on soil carbon density were generally weak.

The variation partitioning analysis revealed that the four categories of factors collectively explained a high proportion of variations in soil carbon mineralization except in the upper soil layer (55% unexplained; Fig. 10B,D,F).The variations in soil carbon mineralization explainable by the shared effect of all the four categories of factors were about similar as in soil carbon density (Fig. 10B,D,F, fraction o). The individual effects of MC, S, M, and T factors on soil carbon mineralization were mostly negligible (Fig. 10B,D,F, fractions a, b, c and d) except the M factor in the middle soil layer (33% explained; Fig. 10D, fraction c). The shared effect of the four categories of factors or combinations of two factors explained a large proportion of the variations in soil carbon mineralization in the upper soil layer, and the shared effect of MC, S and M factors explained 42% and 26% of the variations in soil carbon mineralization in the middle and lower soil layers, respectively (Fig. 10D,F, fraction k). The shared effect of S and M factors also explained a large proportion of the variations in soil carbon mineralization in the lower soil layer (Fig. 10F, fraction f).

Individually, the M factor explained the greatest variations in soil carbon density and mineralization among the four categories of factors. In the 0–5cm soil layer, the effect of the MC factor was weaker than that of the S and T factors in explaining the variations in soil carbon density, and was stronger than that of the S and T factors in explaining the variations in soil carbon mineralization. In the 5–10 cm soil layer, the effect of the MC factor was stronger than that of the S and T factors in explaining the variations in both soil carbon density and soil carbon mineralization. In the 10–20 cm soil layer, the effect of the S factor was stronger than that of the MC and T factors in explaining the variations in soil carbon density and soil carbon mineralization.

**Figure 11:**
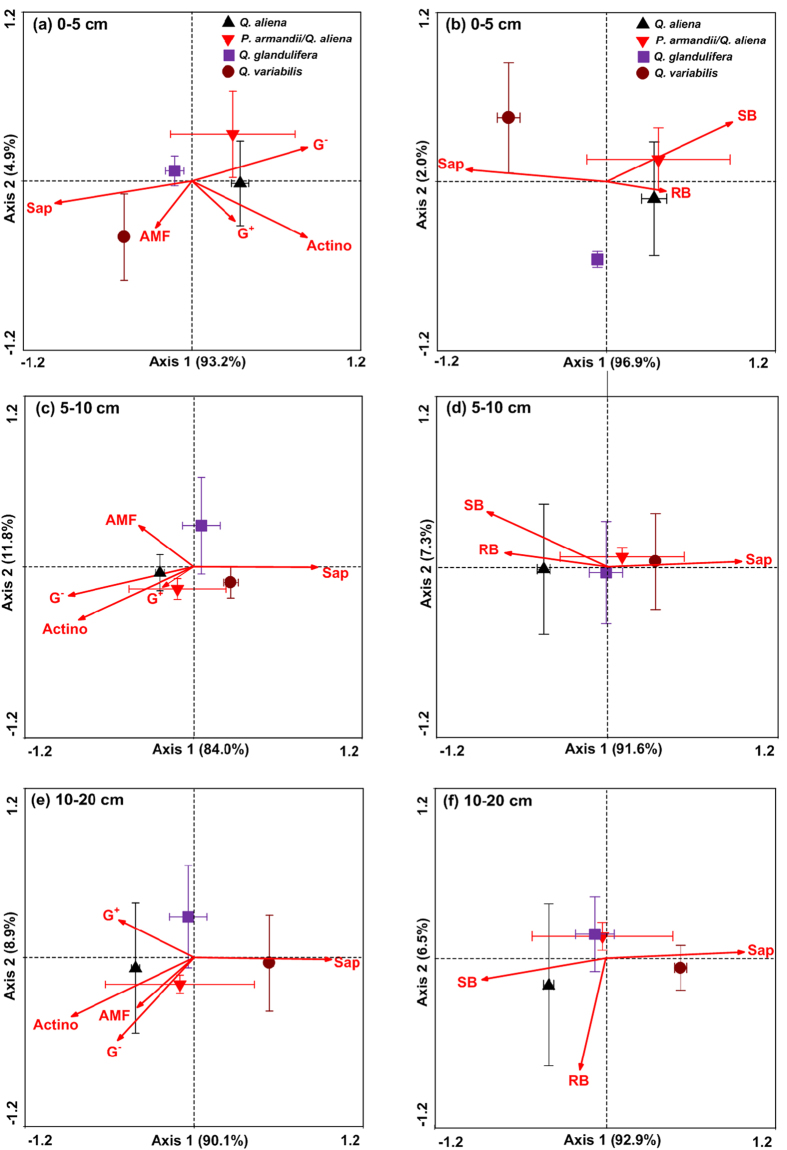
Redundancy analysis (RDA) ordination of microbial community function based on the specific activities of four soil extracellular enzymes (i.e. β-glucosidase, β-N–acetylglucosaminidase, phenol oxidase and peroxidase activities per soil microbial biomass). Arrow length indicates the importance of each soil microbial community types and their relationship with soil microbial community function in plots of *Q. aliena* forest, mixed *P. armandii*/*Q. aliena* forest, *Q. glandulifera* forest, and *Q. variabilis* forest in mid-August 2012. (**a**) Grouping of soil microbial community types by conventional PLFA classification; (**b**) grouping of soil microbial community types based on the carbon sources they feed on. Sap, saprophytic fungi; G^+^, Gram-positive bacteria; G^−^, Gram-negative bacteria; AMF, arbuscular mycorrhizal fungi; Actino, actinomycetes; SB, soil bacteria group; RB, rhizospheric bacteria group. The vertical and horizontal bars show standard errors of means (n = 3).

The relationships between microbial community function, as inferred by microbial-biomass specific enzyme activities (response variables), and the abundance of five soil microbial community types (explanatory variables) are illustrated by the RDA ordination biplots for different forest types (Fig. 11a,c,e). The centroid of forest types occupied different ordination spaces, indicating that the microbial community function was differentiated by forest types. The RDA ordination biplots illustrated inconsistent patterns of associations between microbial community function and microbial community types among forest types. The soil microbial community function in the *Q. aliena* and *P. armandii*/*Q. aliena* plots were positively related to the abundance of Gram-negative bacteria (G^−^) and actinomycetes (Actino), but negatively to the abundance of saprophytic fungi (Sap; Fig. 11a,c,e). However, the soil microbial community function in the *Q. variabilis* plots was positively related to the abundance of Sap, and negatively to the abundance of G^+^, G^−^ and Actino. In the *Q. glandulifera* plots, the soil microbial community function was positively related to the abundance of AMF and Sap in the 0–5 cm and 5–10 cm soil layers, and negatively to the abundance of AMF and Sap in the 10–20 cm soil layer.

The centroid of forest types also occupied different ordination spaces when the soil microbial community types were grouped based on the carbon sources they feed on (Fig. 11b,d,f). The soil microbial community function in the *Q. aliena* plots was positively related to the abundance of soil bacterial group (SB) and rhizospheric bacterial group (RB), and negatively to the abundance of Sap. In contrast, the soil microbial community function in the *Q. variabilis* plots was positively related to the abundance of Sap, and negatively to the abundance of SB and RB. The soil microbial community function in the *P. armandii*/*Q. aliena* and *Q. glandulifera* plots did not display consistent relationships with any of the microbial community types across soil layers.

## Discussion

In forest ecosystems, soil carbon cycle is primarily mediated by biological processes, specifically through the activities of soil micro-organisms^2^,^3^,^30^. Environmental perturbations may influence soil carbon dynamics through modification of soil microbial community structure and function. In this study, we found that soil microbial community structure and function significantly varied across contrasting forest types, and that the variations were apparently related to differences in the quantity and quality of litter, soil physicochemical properties, and microclimate. The higher amounts of microbial biomass carbon and lower microbial metabolic quotient in the *Q. aliena* forest could well be explained by a combination of higher substrate quality^17^. In contrast, the lower amounts of microbial biomass carbon and higher microbial metabolic quotient in the *Q. variabilis* forest were related to lower site fertility (Tables S2 and S3). Our results are supported by findings in other studies that soil carbon cycling is influenced by prevailing site conditions and complex interactions among biotic and abiotic factors^13^,^21^. Therefore, alteration in the characteristics of vegetation could induce profound changes in the below-ground carbon cycling^31^,^32^.

Changes in soil microbial community composition are likely to result in shifts in the functioning of microbial communities, further altering soil biological and physiochemical processes^33^. We found significant differences among the four forest types in soil microbial community structure, which were significantly related to measures of microbial community physiological activities involved in soil carbon metabolisms, suggesting that variations in soil microbial community structure could lead to differential soil microbial functioning in soil carbon transformation and/or turnover among contrasting forest types. Our results show that the nutrient-rich *Q. aliena* forest has greater microbial biomass-specific activities of β-glucosidase (hydrolytic enzyme) and lower activities of phenol oxidase and peroxidase (oxidase enzyme), associated with lower microbial carbon to nitrogen ratio, in clear contrast to the nutrient-poor *Q. variabilis* forest. Bacteria are typically found in soils rich in nutrients and are correlated with the activities of hydrolytic enzymes involved in carbon acquisition, whereas the fungal community trends to increase with decreasing soil fertility and is correlated with the activities of oxidase enzymes involved in degradation of chemically complex compounds (i.e. lignin)^14^,^34^. However, the microbial community function, as indicated by specific activities of soil extracellular enzymes, was not strongly and consistently associated with specific microbial community types across different forest types in our study.

Results in this study suggest the likely deficiency in using microbial community types by the PLFAs method to represent the microbial functional groups. We classified microbial community types based on different PLFAs (i.e. Gram-negative bacteria: 16:1ω7c, cy17:0, 18:1ω7c and cy19:0) and carbon sources they potentially utilize, but some of the PLFAs may come from different functional groups; for example, the PLFAs cy17:0 and cy19:0, which are usually classified as Gram-negative bacteria, sometimes are also considered to be indicators of Gram-positive bacteria^35^. Even though the fatty acid 18:2ω6,9c is common in saprotrophic fungi, it also occurs in some cyanobacteria and soil mosses^36^. The classification based on carbon sources is also fraught with ambiguity of the functional role of specific microbial community types. The PLFA patterns could be considered an integrated measure of all the micro-organisms present in soil irrespective metabolic capacity. Nevertheless, to quantitatively relate microbial community composition to their functioning, we need a better understanding of microbial functional redundancy^3^. Moreover, soil microbial community composition and activities are highly susceptible to changes in environmental conditions^14^, and none of the soil enzyme assay approaches is able to completely reflect the *in situ* conditions of forest soils^37^. Combining molecular approaches with long-term monitoring might be necessary to separate different microbial community functional groups in order to more accurately assess the relationship between microbial community structure and functioning^38^.

We did not detect distinct patterns in microbial carbon to nitrogen ratio, microbial metabolic quotient and specific soil extracellular enzyme activities among stand age classes in the *Q. aliena* forest, suggesting that the soil microbial community structure and function may not have experienced significant shifts with stand development from young to old-growth stages when the forest structures are not much altered. In addition, the *P. armandii*/*Q. aliena* forest and the *Q. aliena* forest were also similar in soil physicochemical properties and microbial community structure and activities. This finding differs from previous studies reporting differentiated soil microbial community structures between contrasting forest types^26^. In clear contrast to the finding that soil microbial community structure in a mixed spruce-birch stand had been predominantly influenced by the coniferous spruce trees^39^, here we found that the broadleaved oak trees primarily shaped the soil microbial properties in the mixed oak-pine forest; the values of microbial variables were mostly similar between the *P. armandii/Q. aliena* forest and the pure *Q. aliena* forests. It should be noted that the *P. armandii* and *Q. aliena* forest stands were formed by planting *P. armandii* trees on originally the *Q. aliena* forest site in the study area^14^. It is therefore probable that the “home range advantage” plays a role in determining the soil microbial properties in artificially established mixed-wood forests.

The different forest types used in this study occur in a relatively narrow geographical range with similar development history. Despite a common locality and similar topography, soil microbial biomass and community structure, microbial biomass-specific extracellular enzyme activities and most of the selective site factors varied significantly across contrasting forest types, but the associations between specific activities of soil extracellular enzymes and specific microbial community types were generally weak and inconsistent across forest types, implicating the existence of a complex mechanism in the microbial regulation of soil carbon metabolism in relation to differences in the occurrence and dominance of forest tree species.

Previously using the approach of structure equation modelling (SEM) and based on data of single sampling in mid-August 2012 in the same study, we identified dominant biotic and aibiotic factors in affecting soil microbial community structures and consequently soil carbon by pooling data of different forest types and stand age classes^14^. In this study, by including data of multiple sampling during growing seasons of 2011 and 2012 and employing the variation partitioning analysis, we found that the individual effects of the categorical factors of microclimate, soil, microbial traits and tree species in explaining the variations in soil carbon density and mineralization are much weaker than the shared effects of the factors investigated. This suggests interactive effects of site, time and plant community on carbon processes in forest soils and highlights the complex mechanisms of the forest type effects on soil carbon. The significant interactive effects between forest type and time of sampling on soil microbial and enzymatic variables demonstrate the complexity of controls on soil carbon dynamics in space and time concerning variations in plant community composition and structure. The multiple constraints and the complexity in interactions and autocorrelations among various biotic and abiotic factors may exacerbate or offset the effects of individual controlling factors, potentially leading to nonlinearity of the effects of forest types on soil carbon dynamics. Therefore, an explicit understanding on the interplays of biotic and abiotic factors in regulating soil carbon processes is crucial for sensibly predicting how soil carbon cycling would respond to climate change with alteration of vegetation type and plant community structure across the landscapes and regions^40^.

## Conclusion

Our findings point to differential controls on soil carbon density and mineralization among contrasting forest types and complex interplays among various biotic and environmental factors in regulating soil carbon dynamics. This imposes a challenge to incorporate microbial processes for constraining soil carbon dynamics in global carbon cycle models.

## Materials and Methods

### Study site

The study was located in the Baotianman Long-Term Forest Ecosystem Research Station in the Baotianman Nature Reserve (latitude 33°35′43′′-33°20′12′′N, longitude 111°46′55′′-112°03′32′′E, and elevation 600–1860 m), in the east of the Qinling Mountain Range in central China. The region is in a transition zone from warm temperate to northern subtropical climate, characterized as of a continental eastern monsoon type, with annual mean air temperature of 15.1 °C and mean annual precipitation of 900 mm^19^. Precipitation occurs mainly in the summer months of June through August (55–62%). The soils are dystric cambisols^41^ developed on weathered arenites. The zonal vegetation at the study sites is typically deciduous broadleaved forests dominated by, with changes in topography and position on slopes, *Q. aliena* var. *acuteserrata*, *Q. glandulifera* var. *brevipetiolata*, and *Q. variabilis*, respectively, in the canopy layer. *P. armandii* plantations were established in the region for timber production around 1956, but some stands developed into mixed forests due to natural regeneration of *Q. aliena* var. *acuteserrata*^14^.

### Experimental design

Four contrasting forest types, namely pure *Q. aliena* var. *acuteserrata* (referred as *Q. aliena*), *Q. glandulifera* var. *brevipetiolata* (referred as *Q. glandulifera*), and *Q. variabilis* forests, respectively, and mixed *P. armandii* and *Q. aliena* (referred as *P. armandii*/*Q. aliena*) forests, as well as an age sequence of pure *Q. aliena* forests (~40, ~80, and >160 yrs), were included in this study. The stands of similar age (~80 yrs) were selected for forest types representing the pure *Q. aliena*, *Q. glandulifera*, and *Q. variabilis* forests. The *P. armandii/Q. aliena* forest stands were aged >50 yrs. Stand age was obtained from forest management records and tree-ring samples. All sites are maintained under natural conditions and have not experienced apparent anthropogenic disturbance in recent history.

In April 2011, three 20 m × 20 m plots were established for each of the four forest types and the age sequence stands of *Q. aliena* forests. All plots were located in separate forest stands spatially distinguished by catchments and slopes, with a minimum distance of 300 m between the adjacent plots. Field measurements were made in each plot of tree species composition and stand structure, descriptive site characteristics, and soil physicochemical properties.

### Field measurements and sampling

Soil temperature at 10 cm depth (T_soil10_) was measured on each plot using HOBO Data Loggers (TidbiT v2 Temp, Onset Computer Corporation, USA) at 30 min intervals. We set out ten litter traps on each plot for measuring litter_fall_. Each trap was 0.56 m^2^ and made of 1 mm mesh nylon netting fixed on a PVC frame. All litter traps were placed 0.5 m above the forest floor and supported by PVC sticks. Forest floor litter samples (including undecomposed plant tissues, partially decomposed duff, and O_e_ and O_a_ horizons) were also collected on each plot at 12 random locations within a 20 cm diameter ring-frame, and oven-dried at 65 °C to constant weight to determine litter_mass_. Twelve root core samples were also collected on each plot by soil layers for 0–5 cm, 5–10 cm, and 10–20 cm depths using a 15 cm inner diameter soil-corer. The roots were washed clean using 0.4 mm mesh bags, and the live roots were separated into coarse- (>5 mm), medium- (2–5 mm) and fine-roots (<2 mm) before being oven-dried at 65 °C to constant weight. The live and dead roots were distinguished following the method of Vogt and Persson (1991)^42^. All litter and live root samples were measured for total dry mass, and analyzed for concentrations of C and N, and the acid soluble fraction and insoluble residue (i.e. the “Klason lignin”)^43^.

Soil samples were collected in June, August and October in 2011, and in April, June, August, and October in 2012, at 12 systematically arranged locations on each plot at a distance of 5 m from the plot center and in the directions of 0, 30, 60, 90, 120, 150, 180, 210, 240, 270, 300, and 330°, respectively. They were collected at depths of 0–5 cm, 5–10 cm and 10–20 cm using a 10 cm inner diameter soil-corer. The samples were mixed to obtain one composite sample for each of the soil layers on each plot. All soil samples were immediately stored on ice in insulated containers upon collection and sieved to pass a 2 mm mesh after returning to laboratory. The processed soil samples were stored in a refrigerator at −20 °C before being further analyzed.

### Measurements of soil physicochemical properties

The gravimetric soil water content, %SWC, was calculated from the mass loss after drying the samples at 105 °C to a constant weight, for at least 48 hrs. We used a hydrometer method for analysis of soil particle size distribution. Soil pH was measured by mixing the soil sample with deionized water at a 1:2.5 ratio (w/v). The supernatants were measured using a pH meter (HI-9125, Hanna Instruments Inc, Woonsocket, USA). SOC content was analyzed by K_2_Cr_2_O_7_-H_2_SO_4_ calefaction method. TN_soil_ was analyzed using Kjeldahl digestion procedure. Soil NH_4_-N and NO_3_-N were determined from 10 g (dry mass) of soil using 50 ml 2 M KCl extraction procedure, and analyzed colorimetrically by a continuous flow analyzer (SEAL AA3, Norderstedt, Germany).

Microbial biomass carbon (MBC) and nitrogen (MBN) were measured by fumigation-extraction method, using 0.5 M K_2_SO_4_ as extracting agent^44^. Dichromate oxidation method and semi-micro Kjeldahl method were used to determine carbon and nitrogen in the extracts, respectively. Dissolved organic carbon (DOC) was determined by directly measuring carbon concentration in the 0.5 M K_2_SO_4_ extract solution without fumigation treatment. The MBC was calculated as:


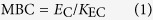


where *E*_C_ is the difference between the organic carbon extracted from fumigated soils and non-fumigated soils, and *Κ*_EC_ is a conversion factor with a given value of 0.38^44^. The MBN was calculated as:


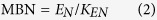


where *E*_*N*_ is the difference between the total nitrogen extracted from fumigated soils and non-fumigated soils, and *Κ*_*EN*_ is a conversion factor with a given value of 0.54^45^.

### Phospholipid fatty acids (PLFAs)

Microbial community structure was assessed by analyzing the composition of extractable ester-linked PLFAs^46^. Concentrations of individual PLFAs were calculated based on 19:0 internal standard concentrations. The indicator PLFAs were used for classification of microbial community types. Bacterial community was represented by PLFAs i14:0, 15:0, i15:0, a15:0, i16:0, 16:1ω7c, 17:0, a17:0, i17:0, cy17:0, 18:1ω7c and cy19:0^44^. Gram-positive bacteria (G^+^) are composed of PLFAs i14:0, i15:0, a15:0, i16:0, a17:0 and i17:0^47^,^48^, Gram-negative bacteria (G^−^) 16:1ω7c, cy17:0, 18:1ω7c and cy19:0^47^, actinomycete (Actino) 10Me16:0, 10Me17:0, 10Me 18:0^13^, saprotrophic fungi (Sap) 18:2ω6,9c^48^, and arbuscular mycorrhizal fungi (AMF) 16:1ω5c^13^,^48^. Other PLFAs, such as 14:0, 16:0, 16:1 2OH, 16:1ω9c, 17:1ω8c, and 18:1ω9c, were also used for analysis of the microbial composition^47^. In addition, we also classified some of the PLFA markers for bacteria, actinomycete (bacteria) and AMF into a rhizospheric bacteria group (16:1ω7c, 18:1ω7c, 16:1ω5c) and a soil bacteria group (i15:0, a15:0, i16:0, 10Me16:0, 10Me18:0, cy17:0, cy19:0) based on the carbon sources they feed on^49^,^50^,^51^.

### Soil extracellular enzyme assays

We assessed four soil extracellular enzymes involved in degrading cellulose (β-1,4-glucosidase [BG]), chitin (β-1,4-N-acetylglucosaminidase [NAG]), and lignin (phenol oxidase [PO] and peroxidase [PER]), respectively. The activities of BG (EC: 3.2.1.21) and NAG (EC: 3.2.1.30) were determined by the conventional *p*-nitrophenol assays^52^,^53^, and PO and peroxidase PER activities were determined using 1-3,4-dihydroxyphenylalanine (L-DOPA) as substrate^54^,^55^. For PO, the reaction mixture was composed of 2 ml 5 mM L-DOPA solution and soil slurry (1 g fresh soil with 1.5 ml 50 mM sodium acetate buffer), and PER activity assays received 2 ml of 5 mM DOPA and soil slurry (1 g fresh soil with 1.5 ml 50 mM sodium acetate buffer), plus 0.2 ml of 0.3% H_2_O_2_. All total enzyme activities were expressed as μmol g^−1^ soil h^−1^, on soil dry weight basis.

### Soil and microbial respiration

Soil CO_2_ efflux was measured during the growing season (April—October) from June 2011 to October 2013 using a Li-8100 soil CO_2_ flux system (LI-COR Inc., NE., USA). The measurements were made on twelve PVC collars on each plot during 10:00–17:00 h over a three-day period every two months starting in April and ending in October each year. The PVC collars on each plot were systematically arranged; specifically, each 20 m × 20 m plot was divided into four 10 m × 10 m subplots, and three PVC collars were placed at a distance of 3 m from each subplot center and in the directions of 0, 120 and 240°, respectively. Soil temperature and volumetric soil water content at 5 cm depth were concurrently measured near each PVC collar. Each PVC collar is at 10 cm in diameter and 5 cm in height, with 3 cm insertion into soil.

We computed the annual soil respiration based on the temperature function of soil respiration^20^.

Microbial respiration, MR, was measured by determining CO_2_ evolution over a 12 day incubation^56^. Specifically, 20 g (dry weight equivalent) of each soil sample was adjusted to 60% of water holding capacity (WHC), and placed in a 250 ml Mason jar. Respired CO_2_ was absorbed in 10 ml 0.1 M NaOH solution suspended inside the jar. All jars were sealed with rubber septum and incubated at 25 °C for 12 days in the dark. Each NaOH solution containing dissolved CO_2_ was then titrated with 0.05 M HCl solution to determine the amount of CO_2_ evolved at 3 day intervals. After each reading the jars were left open for half an hour to ventilate and then re-sealed.

The microbial metabolic quotient, qCO_2_, was calculated by:


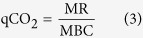


### Data analysis

Differences in plant, site, microclimatic and soil variables (Figs 1 and 2; Tables S1–S5) among forest types/stand age classes were tested by analysis of variance (ANOVA), separately for each soil layer where applicable. Multiple measurements of microbial variables and soil extracellular enzymes over time (Figs 4, 6 and 8 and S1–S4; Tables S6–S8) were examined by repeated measures analysis of variance (RMANOVA). We also used one-way ANOVA to test the effects of forest types/stand age classes on the microbial variables and soil extracellular enzymes for each sampling time (Figs 3, 5, 7 and 9) if a variable was significantly affected by an interaction between forest type/stand age classes and sampling time. All these analyses were performed using SPSS 17.0 for Windows (SPSS Inc., Chicago, USA). The least significant difference (LSD) was used for comparisons of means with a confidence level of *P* < 0.05.

The “Varpart” function in the “Vegan” package was used to partition the variation of soil carbon density (involving both soil carbon concentration and bulk density) and soil carbon mineralization (represented by the specific activities of soil extracellular enzymes. i.e. activity per SOC) by different categories of predictors (i.e. microclimate, soil physiochemical properties, tree species traits, and microbial traits), using redundancy analysis (RDA). We reported the variation explained in each RDA model as the adjusted *R*^*2*^ (*R*^*2*^_adj_), which takes the number of predictor variables and sample size into account to prevent the inflation of *R*^*2*^ values. When a negative *R*^*2*^_adj_ was obtained, we interpreted it as a zero value, meaning that not all fractions of one variation partitioning always add up to a perfect 100%^57^,^58^. These analyses were performed on R 3.0.1 software (R-Development Core Team 2009) with Hellinger-transformed data.

We used Redundancy analysis (RDA) to examine the interrelationships between soil microbial community types and specific activities of soil extracellular enzymes, following the procedures in CANOCO software for Windows 4.5 (Biometris-Plant Research International, Wageningen, Netherlands), based on log-transformed data.

## Additional Information

**How to cite this article**: You, Y.-M. *et al.* Differential controls on soil carbon density and mineralization among contrasting forest types in a temperate forest ecosystem. *Sci. Rep.* **6**, 22411; doi: 10.1038/srep22411 (2016).

## Supplementary Material

Supplementary Tables

Supplementary Figures
