# Supplementary material for: Differential controls on soil carbon density and mineralization among contrasting forest types in a temperate forest ecosystem
Source: Sci Rep. 2016 Mar 1;6:22411. doi: 10.1038/srep22411 (PMC4772072; doi:10.1038/srep22411)
Supplement: Supplementary Tables [file srep22411-s1.pdf]

Supplementary Tables S1-S8

Title: Differential controls on soil carbon density and mineralization among contrasting forest types in a temperate forest ecosystem

Authors: Yeming You[1,2,3], Juan Wang[1,2*], Xiaolu Sun[1,2], Zuoxin Tang[1,2], Zhiyong Zhou[1,2], Osbert Jianxin Sun[1,2]*

[1]College of Forest Science, Beijing Forestry University, Beijing 100083, China, [2]Institute of Forestry and Climate Change Research, Beijing Forestry University, Beijing 100083, China, [3]College of Forestry, Guangxi University, Nanning, Guangxi 530004, China.

Author for correspondence:

Osbert Jianxin Sun

Tel: +86 10 62337095

Email: sunjianx@bjfu.edu.cn

**These authors contributed equally to this work.*

Table S1 Summary of *F*-values and significance level in analysis of variance (ANOVA) evaluating the effects of forest type/stand age class on plant, site and soil variables within given soil layers (Forest type: d.f. = 3, 8; Stand age class: d.f. = 2, 6).

	FR	%SWC	%Sand	%Silt	%Clay	TN_{soil}	NH_4-N	NO_3-N	pH	C/N_{soil}	DOC
Comparison among forest types											
0-5 cm	28.35***	3.00	3.23	2.98	4.04	4.42*	0.72	4.49*	8.20**	12.63**	4.41*
5-10 cm	0.56	2.94	6.22*	3.00	7.03*	7.85**	2.33	1.52	12.03**	3.01	1.30
10-20 cm	7.59*	2.8	5.75*	3.10	4.92*	9.26**	1.23	6.28*	23.95***	13.74**	0.90
Comparison among stand age classes of *Q. aliena* forest											
0-5 cm	1.61	4.81	1.11	1.52	0.60	7.77*	2.59	1.69	0.38	0.21	0.22
5-10 cm	0.32	1.47	0.95	0.12	1.47	0.15	0.14	0.05	0.96	0.50	0.08
10-20 cm	1.04	2.05	1.76	0.09	1.20	1.08	0.42	1.99	3.16	0.46	0.35

Note: *$P < 0.05$; **$P < 0.01$; ***$P < 0.001$.FR, fine root mass; %SWC, gravimetric soil water content (w/w); %Sand, percentage of sand; %Silt, percentage of silt; %Clay, percentage of clay; TN_{soil}, soil total nitrogen; NH_4-N, ammonium nitrogen; NO_3-N, nitrate nitrogen; pH, soil pH value; C/N_{soil}, soil carbon to nitrogen ratio; DOC, dissolved organic carbon.

Table S2 Values of site, microclimatic and plant variables for comparison among contrasting forest types (means ± standard errors; n = 3).

	Forest types				ANOVA	
	Q. aliena	*P. armandii/ Q. aliena*	*Q. glandulifera*	*Q. variabilis*	*F*	*P*
BA	42.1 ±3.7a	43.9 ±4.2a	44.8 ±4.1a	48.0 ±2.6a	0.45	0.725
Slope (%)	41.1 ±3.0a	15.0 ±10.7b	24.6 ±8.0ab	25.6 ±2.2ab	2.43	0.140
T_{soil10} (℃)	11.4 ±0.4bc	11.1 ±0.3c	12.0 ±0.1b	13.1 ±0.1a	13.79	0.002
$Litter_{mass}$	4563.6 ±569.3ab	5133.7 ±503.0a	3956.6 ±663.6ab	3012.6 ±432.8b	2.73	0.114
$Litter_{fall}$	687.1 ±13.9a	514.9 ±46.7b	382.5 ±16.0c	456.6 ±9.8c	24.73	< 0.001
$\%N_{litter}$	1.4 ±0.1a	1.1 ±0.1b	1.1 ±0.1b	0.9 ±0.0b	7.56	0.010
C/N_{litter}	35.3 ±0.5b	47.8 ±5.3a	45.5 ±5.4ab	53.3 ±5.3a	4.39	0.042
$\%Lignin_{litter}$	29.1 ±2.8a	28.8 ±4.8a	28.0 ±3.4a	25.3 ±0.6a	0.37	0.775
$Lignin/N_{litter}$	20.9 ±1.7b	24.9 ±2.4ab	26.6 ±0.6a	27.9 ±2.0a	4.43	0.041
$\%Lignin_{root}$	21.5 ±0.2b	44.0 ±4.6a	34.2 ±1.1ab	28.8 ±5.3b	7.2	0.011
$Lingin/N_{root}$	30.8 ±4.7b	58.5 ±5.2a	45.9 ±1.7ab	34.6 ±8.1b	5.29	0.027
C/N_{root}	70.2 ±3.9a	58.7 ±3.2a	67.3 ±5.4a	71.2 ±5.1a	1.7	0.244

Values designated by different letters for a given variable are significantly different among forest types ($P < 0.05$). BA, stem basal area (m^2 ha^{-1}); Slope, site slope; T_{soil10}, soil temperature at 10 cm depth; $Litter_{mass}$, forest floor litter mass (g m^{-2}); $Litter_{fall}$, annual litterfall (g m^{-2} yr^{-1}); $\%C_{litter}$, litter carbon content in percentage; $\%N_{litter}$, litter nitrogen content in percentage; C/N_{litter}, litter carbon to nitrogen ratio; $\%Lignin_{litter}$, litter lignin content in percentage; $Lignin/N_{litter}$, litter lignin to nitrogen ratio; $\%Lignin_{root}$, root lignin content in percentage; $Lingin/N_{root}$, root lignin to nitrogen ratio; C/N_{root}, root carbon to nitrogen ratio.

Table S3 Values of plant, microclimatic and soil variables for comparison among contrasting forest types (means ± standard errors; n = 3).

	0 – 5 cm soil layer				5 – 10 cm soil layer				10 – 20 cm soil layer			
	Q. aliena	*P. armandii / Q. aliena*	*Q. glandulifera*	*Q. variabilis*	*Q. aliena*	*P. armandii / Q. aliena*	*Q. glandulifera*	*Q. variabilis*	*Q. aliena*	*P. armandii / Q. aliena*	*Q. glandulifera*	*Q. variabilis*
FR	3.7 ±0.5bc	2.7 ±0.2c	4.15 ±0.3b	5.5 ±0.2a	2.9 ±0.5a	3.1 ±0.7a	3.5 ±0.3a	3.4 ±0.4a	1.5 ±0.1a	1.0 ±0.1b	1.7 ±0.1a	1.8 ±0.1a
%SWC	38.5 ±2.7ab	42.8 ±10.1a	37.0 ±2.0ab	21.6 ±0.4b	31.3 ±2.7a	32.9 ±9.0a	28.2 ±1.8ab	14.8 ±0.1 b	28.4 ±2.3ab	30.7 ±8.1a	25.5 ±2.4ab	14.1 ±0.3b
%Sand	61.7 ±3.8b	63.0 ±7.8b	62.3 ±1.9b	78.3 ±0.7a	68.7 ±4.5b	69.3 ±5.5ab	57.7 ±0.7b	79.3 ±0.3a	64.3 ±0.9b	74.0 ±5.5ab	65.3 ±1.7b	79.0 ±0.6a
%Silt	10.7 ±1.7ab	9.0 ±1.5b	13.7 ±0.3a	8.7 ±1.3b	11.3 ±2.3ab	8.0 ±1.0b	13.7 ±1.5a	9.0 ±0.0ab	11.4 ±1.5a	6.7 ±1.2b	10.0 ±1.0ab	9.7 ±0.7ab
%Clay	27.6 ±2.2a	28.0 ±6.4a	24.0 ±1.5ab	13.0 ±1.2b	20.0 ±2.3ab	22.7 ±4.5a	28.6 ±1.7a	11.7 ±0.3b	24.3 ±2.2a	19.3 ±4.8ab	24.7 ±1.9a	11.3 ±0.3b
TN$_{soil}$	4.69 ±0.1a	3.51 ±1.2ab	2.69 ±0.2b	2.37 ±0.1b	2.41 ±0.2a	1.51 ±0.3b	1.57 ±0.2b	1.00 ±0.0b	1.99 ±0.1a	1.15 ±0.3b	0.94 ±0.0b	0.70 ±0.0b
NH_4-N	48.5 ±3.6a	49.1 ±11.5a	49.3 ±6.2a	39.6 ±0.9a	31.3 ±3.1a	34.3 ±8.1a	34.0 ±0.7a	20.2 ±0.3a	24.4 ±2.9a	27.4 ±8.1a	21.0 ±1.4a	16.1 ±0.1a
NO_3-N	2.6 ±0.4ab	2.9 ±1.1a	1.0 ±0.1c	1.2 ±0.2bc	1.7 ±0.6a	2.2 ±0.9a	0.9 ±0.1a	0.9 ±0.1a	1.7 ±0.1a	1.8 ±0.4a	0.9 ±0.1b	0.8 ±0.0b
pH	4.6 ±0.1a	4.6 ±0.1a	4.2 ±0.1b	4.2 ±0.0b	4.5 ±0.1 a	4.5 ±0.1a	4.2 ±0.0b	4.2 ±0.0b	4.6 ±0.0a	4.6 ±0.0a	4.3 ±0.1b	4.3 ±0.0b
C/N$_{soil}$	13.5 ±0.3c	15.5 ±0.8b	17.4 ±0.6a	17.7 ±0.2a	13.9 ±0.2b	15.8 ±1.0a	15.3 ±0.2ab	16.1 ±0.6a	13.8 ±0.6b	14.9 ±0.2b	16.0 ±0.4ab	16.5 ±0.6a
DOC	149.0 ±22.8b	198.7 ±11.4ab	254.9 ±27.6a	190.1 ±17.7ab	157.7 ±17.3a	177.1 ±5.7a	216.0 ±16.9a	183.6 ±34.6a	144.7 ±17.7a	153.4 ±20.6a	183.6 ±28.3a	179.3 ±9.4a

Values designated by different letters for a given variable are significantly different among forest types within given soil layers ($P < 0.05$). FR, fine root mass (g kg^{-1} soil); %SWC, gravimetric soil water content (w/w); %Sand, percentage of sand; %Silt, percentage of silt; %Clay, percentage of clay; TN$_{soil}$, soil total nitrogen (g kg^{-1} soil); NH_4-N, ammonium nitrogen (mg kg^{-1} soil); NO_3-N, nitrate nitrogen (mg kg^{-1} soil); pH, soil pH value; C/N$_{soil}$, soil carbon to nitrogen ratio; DOC, dissolved organic carbon (mg kg^{-1} soil).

Table S4 Values of microclimatic and plant variables and summary of ANOVA for comparison among contrasting stand age classes of the *Q. aliena* forest (means ± standard errors; n = 3).

	Stand age class			ANOVA	
	40-yrs stand	80-yrs stand	160-yrs stand	*F*	*P*
BA	44.6±3.8a	42.1±3.7a	46.8±2.6a	0.47	0.645
Slope (%)	42.9±8.1a	41.1±3.0a	40.5±3.1a	0.06	0.944
T_{soil10} (℃)	11.8±0.1a	11.4±0.4a	11.1±0.1a	2.22	0.190
$Litter_{mass}$	5534.8±759.4a	4563.6±569.3a	5753.9±566.5a	0.99	0.426
$Litter_{fall}$	655.5±183.7a	687.1±13.9a	627.9±61.2a	0.07	0.933
$\%N_{litter}$	1.3±0.1a	1.4±0.1a	1.4±0.1a	0.32	0.740
C/N_{litter}	37.9±1.6a	35.3±0.5a	37.7±3.3a	0.72	0.526
$\%Lignin_{litter}$	27.8±5.4a	29.1±2.8a	29.6±1.0a	0.04	0.963
$Lignin/N_{litter}$	21.1±4.3a	20.9±1.7a	21.9±0.7a	0.02	0.981
$\%Lignin_{root}$	25.3±2.2a	21.5±0.2a	26.0±4.4a	0.71	0.529
$Lingin/N_{root}$	32.7±1.4a	30.8±4.7a	39.7±2.8a	2.06	0.209
C/N_{root}	77.4±5.4a	70.2±3.9a	73.5±6.3a	0.47	0.645

Values designated by different letters for a given variable are significantly different among stand age classes ($P < 0.05$). BA, stem basal area ($m^2 \cdot ha^{-1}$); Slope, site slope; T_{soil10}, soil temperature at 10 cm depth; $Litter_{mass}$, forest floor litter mass ($g \cdot m^{-2}$); $Litter_{fall}$, annual litterfall ($g \cdot m^{-2} \cdot yr^{-1}$); $\%C_{litter}$, litter carbon content in percentage; $\%N_{litter}$, litter nitrogen content in percentage; C/N_{litter}, litter carbon to nitrogen ratio; $\%Lignin_{litter}$, litter lignin content in percentage; $Lignin/N_{litter}$, litter lignin to nitrogen ratio; $\%Lignin_{root}$, root lignin content in percentage; $Lingin/N_{root}$, root lignin to nitrogen ratio; C/N_{root}, root carbon to nitrogen ratio.

Table S5 Values of plant, microclimatic and soil variables for comparison among contrasting stand age classes of the *Q. aliena* forest (means ± standard errors; n = 3).

	0 - 5 cm soil layer			5 - 10 cm soil layer			10 - 20 cm soil layer		
	40-yrs stand	80-yrs stand	160-yrs stand	40-yrs stand	80-yrs stand	160-yrs stand	40-yrs stand	80-yrs stand	160-yrs stand
FR	3.7 ±0.5a	2.7 ±0.2a	3.8 ±0.6a	2.9 ±0.5a	2.8 ±0.1a	3.1 ±0.1a	1.5 ±0.1a	1.3 ±0.1a	1.2 ±0.2a
%SWC	41.8 ±0.6ab	38.5 ±2.7b	47.4 ±2.2a	34.6 ±1.1a	31.3 ±2.7a	36.7 ±2.6a	33.3 ±1.8a	28.4 ±2.3a	31.3 ±0.6a
%Sand	57.3 ±1.8a	61.6 ±3.8a	56.7 ±1.5a	60.3 ±5.7a	68.7 ±4.5a	64.3 ±1.5a	65.6 ±0.3a	64.3 ±0.9a	63.3 ±1.2a
%Silt	12.7 ±1.7a	10.7 ±1.7a	14.0 ±0.0a	11.0 ±0.0a	11.3 ±2.3a	12.0 ±1.0a	11.7 ±0.7a	11.4 ±1.5a	11.0 ±1.2a
%Clay	30.0 ±0.6a	27.7 ±2.2a	29.3 ±1.5a	28.7 ±5.7a	20.0 ±2.3a	23.7 ±0.9a	22.7 ±0.9a	24.3 ±2.2a	25.7 ±0.3a
TN_{soil}	4.2 ±0.0b	4.7 ±0.1a	4.5 ±0.1a	2.3 ±0.3a	2.4 ±0.2a	2.4 ±0.1a	1.8 ±0.1a	2.0 ±0.1a	2.1 ±0.2a
NH_4-N	51.8 ±2.6a	48.5 ±3.6a	59.7 ±6.2a	33.7 ±4.3a	31.3 ±3.1a	32.6 ±2.6a	27.6 ±3.6a	24.4 ±2.9a	24.5 ±1.7a
NO_3-N	3.3 ±0.3a	2.7 ±0.4a	3.4 ±0.4a	1.8 ±0.4a	1.7 ±0.6a	2.0 ±0.8a	1.6 ±0.2a	1.7 ±0.1a	1.9 ±0.1a
pH	4.6 ±0.1a	4.6 ±0.1a	4.5 ±0.1a	4.5 ±0.1a	4.5 ±0.1a	4.4 ±0.1a	4.7 ±0.0a	4.6 ±0.0a	4.6 ±0.1a
C/N_{soil}	13.6 ±0.2a	13.5 ±0.3a	13.4 ±0.2a	13.5 ±0.5a	13.9 ±0.2a	13.7 ±0.2a	14.4 ±0.3a	13.8 ±0.6a	13.6 ±0.8a
DOC	164.2 ±21.3a	149.0 ±22.8a	166.3 ±15.6a	168.5 ±23.4a	157.7 ±17.3a	157.7 ±25.5a	166.3 ±11.4a	144.7 ±17.7a	155.5 ±23.4a

Values designated by different letters for a given variable are significantly different among stand age classes within given soil layers (P < 0.05). FR, fine root mass (g ⋅kg^{-1} soil); %SWC, gravimetric soil water content (w/w); %Sand, percentage of sand; %Silt, percentage of silt; %Clay, percentage of clay; TN_{soil}, soil total nitrogen (g ⋅kg^{-1} soil); NH_4-N, ammonium nitrogen (mg ⋅kg^{-1} soil); NO_3-N, nitrate nitrogen (mg ⋅kg^{-1} soil); pH, soil pH value; C/N_{soil}, soil carbon to nitrogen ratio; DOC, dissolved organic carbon (mg ⋅kg^{-1} soil).

Table S6 Summary of *F*-values and significance level in repeated measures analysis of variance (RMANOVA) evaluating the effects of forest type/stand age class (between subjects) and sampling time (within subjects) on microbial biomass C (MBC), microbial biomass N (MBN), microbial C to N ratio (C_{mic}/N_{mic}), microbial respiration (MR), and microbial metabolic quotient (qCO_2) within given soil layers.

		Comparison among forest types						Comparison among stand age classes of *Q. aliena* forest					
		Forest type (*FT*)		Sampling time (*ST*)		*FT* × *ST*		Stand age (*SA*)		Sampling time (*ST*)		*SA* × *ST*	
		d.f.	F	d.f.	F	d.f.	F	d.f.	F	d.f.	F	d.f.	F
0-5 cm	MBC	(3,8)	6.91*	(6,48)	4.80**	(18,48)	1.02	(2,6)	1.20	(6,36)	14.52***	(12,36)	1.39
	MBN	(3,8)	12.27**	(6,48)	7.63***	(18,48)	1.75	(2,6)	1.55	(6,36)	4.39**	(12,36)	0.55
	C_{mic}/N_{mic}	(3,8)	5.71*	(6,48)	5.32***	(18,48)	0.62	(2,6)	1.24	(6,36)	4.43**	(12,36)	0.87
	MR	(3,8)	1.73	(1.71,13.69)	5.60*	(5.13,13.69)	1.06	(2,6)	3.98	(2.25,13.50)	15.93***	(4.50,13.50)	0.72
	qCO_2	(3,8)	5.37*	(6,48)	1.56	(18,48)	0.98	(2,6)	0.03	(2.38,14.27)	2.92*	(4.76,14.27)	1.40
5-10 cm	MBC	(3,8)	7.72*	(2.64,21.15)	6.43***	(7.92,21.15)	3.98**	(2,6)	9.15*	(6,36)	7.64***	(12,36)	3.16**
	MBN	(3,8)	7.35*	(6,48)	1.97	(18,48)	0.93	(2,6)	0.40	(2.64,15.85)	0.55	(5.28,15.85)	0.85
	C_{mic}/N_{mic}	(3,8)	4.81*	(3.06,24.49)	1.24	(9.18,24.49)	1.60	(2,6)	0.26	(2.35,14.12)	1.07	(4.70,14.12)	1.76
	MR	(3,8)	4.60*	(2.01,16.05)	7.76**	(6.03,16.05)	4.76**	(2,6)	0.97	(2.20,13.22)	14.73***	(4.40,13.22)	1.02
	qCO_2	(3,8)	5.39*	(6,48)	3.32**	(18,48)	2.18*	(2,6)	1.06	(6,36)	2.24	(12,36)	1.70
10-20 cm	MBC	(3,8)	5.24*	(2.11,16.89)	0.81	(6.33,16.89)	1.76	(2,6)	1.53	(2.37,14.20)	2.67*	(4.74,14.20)	2.73*
	MBN	(3,8)	6.16*	(6,48)	0.78	(18,48)	1.06	(2,6)	0.26	(6,36)	1.97	(12,36)	0.57
	C_{mic}/N_{mic}	(3,8)	0.93	(2.04,16.32)	0.51	(6.12,16.32)	1.12	(2,6)	0.71	(6,36)	1.42	(12,36)	1.67
	MR	(3,8)	9.03**	(1.62,12.97)	14.94**	(4.86,12.97)	4.94*	(2,6)	0.55	(6,36)	3.47**	(12,36)	0.35
	qCO_2	(3,8)	4.38*	(6,48)	3.15*	(18,48)	1.71	(2,6)	0.48	(2.75,16.48)	3.01	(5.50,16.48)	1.75

Note: Greenhouse-Geisser correction was applied when the sphericity assumption was violated. *P< 0.05; **P< 0.01; *** P< 0.001.

Table S7 Summary of *F*-values and significance level in RMANOVA evaluating the effects of forest type/stand age class (between subjects) and sampling time (within subjects) on total activities of β-1,4-glucosidase (BG), β-1,4-N-acetylglucosaminidase (NAG), phenol oxidase (PO) and peroxidase (PER) within given soil layers.

		Comparison among forest types						Comparison among stand age classes of *Q. aliena* forest					
		Forest type (*FT*)		Sampling time (*ST*)		*FT* × *ST*		Stand age (*SA*)		Sampling time (*ST*)		*SA* × *ST*	
		d.f.	F	d.f.	F	d.f.	F	d.f.	F	d.f.	F	d.f.	F
0-5 cm	BG	(3,8)	9.03**	(6,48)	1.75	(18,48)	1.32	(2,6)	4.73	(6,36)	2.06	(12,36)	0.92
	NAG	(3,8)	4.30*	(2.30,18.43)	5.81**	(6.90,18.43)	2.70*	(2,6)	2.74	(6,36)	5.44***	(12,36)	1.23
	PO	(3,8)	3.46	(3.16,25.27)	8.98***	(9.48,25.27)	2.15	(2,6)	1.06	(6,36)	6.88***	(12,36)	1.13
	PER	(3,8)	2.09	(2.27,18.12)	4.34*	(6.80,18.12)	0.90	(2,6)	0.03	(2.50,14.98)	7.55**	(5.00,14.98)	0.16
5-10 cm	BG	(3,8)	4.75*	(6,48)	8.13***	(18,48)	0.72	(2,6)	1.34	(1.91,11.47)	5.84*	(3.82,11.47)	0.39
	NAG	(3,8)	0.93	(1.75,14.01)	2.66	(5.25,14.01)	0.66	(2,6)	2.15	(1.49,8.93)	1.58	(2.98,8.93)	1.15
	PO	(3,8)	4.37*	(2.76,22.07)	36.71***	(8.28,22.07)	2.76*	(2,6)	2.45	（1.93,11.57）	5.16*	(3.86,11.57)	1.73
	PER	(3,8)	5.74*	(2.30,18.40)	2.65*	(6.90,18.40)	0.56	(2,6)	5.69*	(1.96,11.75)	4.34	(3.92,11.75)	0.41
10-20 cm	BG	(3,8)	5.46*	(6,48)	12.20***	(18,48)	1.78	(2,6)	0.12	(1.00,6.01)	0.81	(2.00,6.01)	0.99
	NAG	(3,8)	4.31*	(1.69,13.53)	6.12*	(5.07,13.53)	1.70	(2,6)	0.19	(1.49,8.94)	1.82	(2.98,8.94)	0.54
	PO	(3,8)	4.27*	(2.73,21.84)	4.39*	(8.19,21.84)	2.43*	(2,6)	1.69	(2.15,12.89)	6.90**	(4.30,12.89)	0.29
	PER	(3,8)	4.45*	(2.87,22.98)	1.45	(8.61,22.98)	1.45	(2,6)	2.90	(2.09,12.56)	1.99	(4.18,12.56)	0.89

Note: Greenhouse-Geisser correction was applied when the sphericity assumption was violated. *P < 0.05; **P < 0.01; *** P < 0.001.

Table S8 Summary of *F*-values and significance level in RMANOVA evaluating the effects of forest type/stand age class (between subjects) and sampling time (within subjects) on microbial biomass-specific activities of β-1,4-glucosidase (BG), β-1,4-N-acetylglucosaminidase (NAG), phenol oxidase (PO) and peroxidase (PER) within given soil layers.

		Comparison among forest types						Comparison among stand age classes of *Q. aliena* forest					
		Forest type (*FT*)		Sampling time (*ST*)		*FT* × *ST*		Stand age (*SA*)		Sampling time (*ST*)		*SA* × *ST*	
		d.f.	F	d.f.	F	d.f.	F	d.f.	F	d.f.	F	d.f.	F
0-5 cm	BG	(3,8)	8.01**	(2.75,22.00)	4.99*	(8.25,22.00)	0.75	(2,6)	0.76	(6,36)	4.11**	(12,36)	0.77
	NAG	(3,8)	4.54*	(6,48)	6.29***	(18,48)	2.55**	(2,6)	0.26	(1.83,10.97)	4.33*	(3.66,10.97)	0.99
	PO	(3,8)	15.38**	(6,48)	4.32**	(18,48)	1.11	(2,6)	2.17	(6,36)	10.67***	(12,36)	1.22
	PER	(3,8)	16.32**	(6,48)	4.19**	(18,48)	0.89	(2,6)	0.27	(6,36)	8.10***	(12,36)	0.38
5-10 cm	BG	(3,8)	4.74*	(3.38,27.04)	7.47**	(10.14,27.04)	1.83	(2,6)	0.15	(6,36)	9.16***	(12,36)	0.97
	NAG	(3,8)	1.05	(1.29,10.35)	2.63	(3.87,10.35)	0.48	(2,6)	3.17	(6,36)	2.93*	(12,36)	1.55
	PO	(3,8)	10.17**	(1.69,13.48)	2.85	(5.07,13.48)	2.79	(2,6)	1.15	(6,36)	3.25*	(12,36)	1.57
	PER	(3,8)	17.65**	(1.44,11.53)	2.28	(4.32,11.53)	1.22	(2,6)	1.09	(6,36)	6.25***	(12,36)	1.00
10-20 cm	BG	(3,8)	4.23*	(6,48)	6.62***	(18,48)	1.31	(2,6)	0.11	(1.00,6.03)	0.74	(2.00,6.03)	0.98
	NAG	(3,8)	1.21	(6,48)	6.14***	(18,48)	2.32*	(2,6)	0.35	(1.62,9.71)	3.33	(3.24,9.71)	1.08
	PO	(3,8)	4.22*	(2.36,18.89)	4.31*	(7.08,18.89)	2.59*	(2,6)	0.71	(1.52,9.10)	2.85	(3.04,9.10)	0.57
	PER	(3,8)	8.83**	(2.63,21.03)	0.79	(7.89,21.03)	0.86	(2,6)	1.48	(1.96,11.75)	2.08	(3.92,11.75)	1.08

Note: Greenhouse-Geisser correction was applied when the sphericity assumption was violated. *P < 0.05; **P < 0.01; *** P < 0.001.
