# Supplementary material for: Differential controls on soil carbon density and mineralization among contrasting forest types in a temperate forest ecosystem
Source: Sci Rep. 2016 Mar 1;6:22411. doi: 10.1038/srep22411 (PMC4772072; doi:10.1038/srep22411)

Supplementary Figures S1-S4

Title: Differential controls on soil carbon density and mineralization among contrasting forest types in a temperate forest ecosystem

Authors: Yeming You[1,2,3], Juan Wang[1,2*], Xiaolu Sun[1,2], Zuoxin Tang[1,2], Zhiyong Zhou[1,2], Osbert Jianxin Sun[1,2]*

[1]College of Forest Science, Beijing Forestry University, Beijing 100083, China, [2]Institute of Forestry and Climate Change Research, Beijing Forestry University, Beijing 100083, China, [3]College of Forestry, Guangxi University, Nanning, Guangxi 530004, China.

Author for correspondence:

Osbert Jianxin Sun

Tel: +86 10 62337095

Email: sunjianx@bjfu.edu.cn

**These authors contributed equally to this work.*

Figure S1 Mean values of microbial biomass N (MBN) and microbial C to N ratio (C/N_{mic}) in 0-5, 5-10, 10-20 cm soil layers in plots of contrasting stand age classes of *Q. aliena* forest during the growing seasons from 2011 to 2012. Error bars represent 1 SE (n = 3). Same letters above the error bars indicate no significant differences among stand age classes within given soil layers ($P > 0.05$).

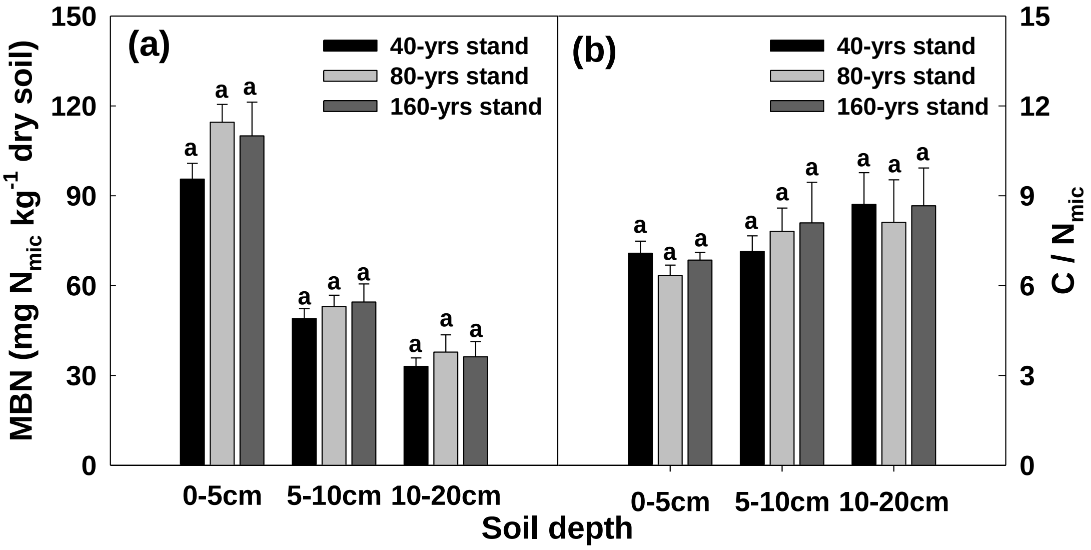

Figure S2 Mean values of soil microbial respiration (MR) and microbial metabolic quotient (qCO_2) in the 0-5, 5-10 and 10-20 cm soil layers in plots of contrasting stand age classes of *Q. aliena* forest during the growing seasons from 2011 to 2012. Error bars represent 1 SE (n = 3). Same letters above the error bars indicate no significant differences among stand age classes within given soil layers ($P > 0.05$).

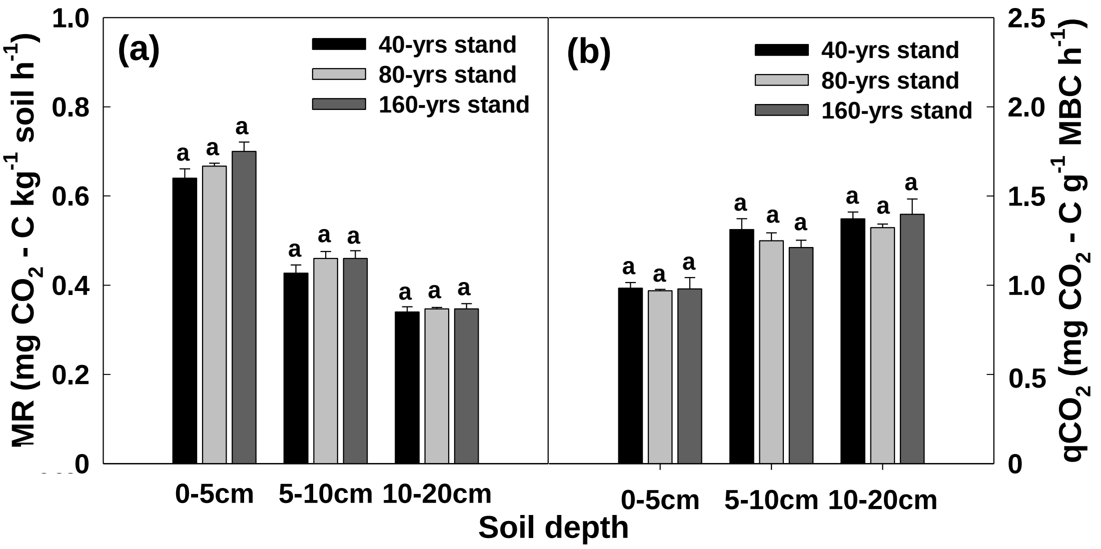

Figure S3 Mean values of activities of β-1,4-glucosidase (BG), β-1,4-N-acetylglucosaminidase (NAG), phenol oxidase (PO) and peroxidase (PER) in the 0-5, 5-10, 10-20 cm soil layers in plots of contrasting stand age classes of *Q. aliena* forest during the growing seasons from 2011 to 2012. Error bars represent 1 SE (n = 3). Different letters above the error bars indicate significant differences among stand age classes within given soil layers ($P < 0.05$).

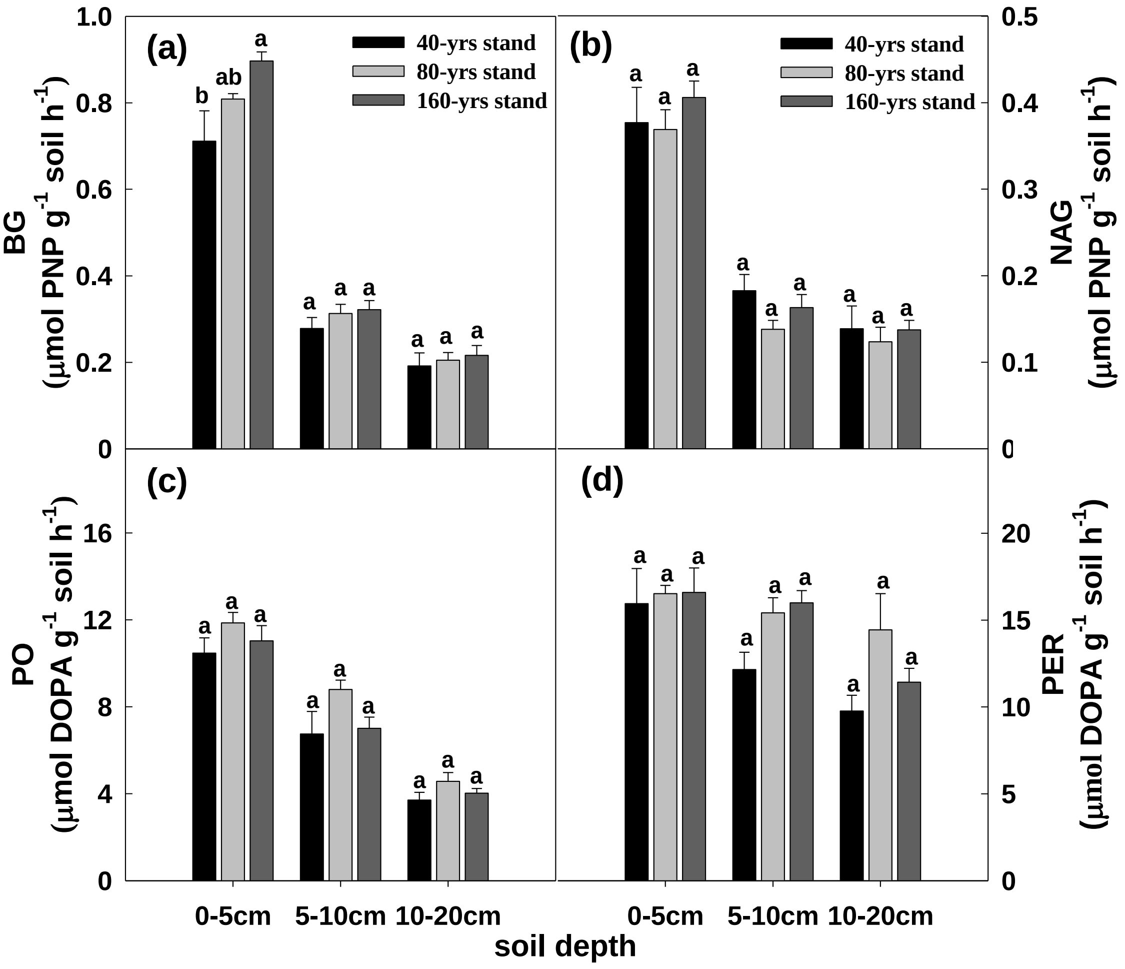

Figure S4 Mean values of specific activities of β-1,4-glucosidase (BG), β-1,4-N-acetylglucosaminidase (NAG), phenol oxidase (PO) and peroxidase (PER) in the 0-5, 5-10, 10-20 cm soil layers in plots of contrasting stand age classes of *Q. aliena* forest during the growing seasons from 2011 to 2012. Error bars represent 1 SE (n = 3). Same letters above the error bars indicate no significant differences among stand age classes within given soil layers ($P > 0.05$).

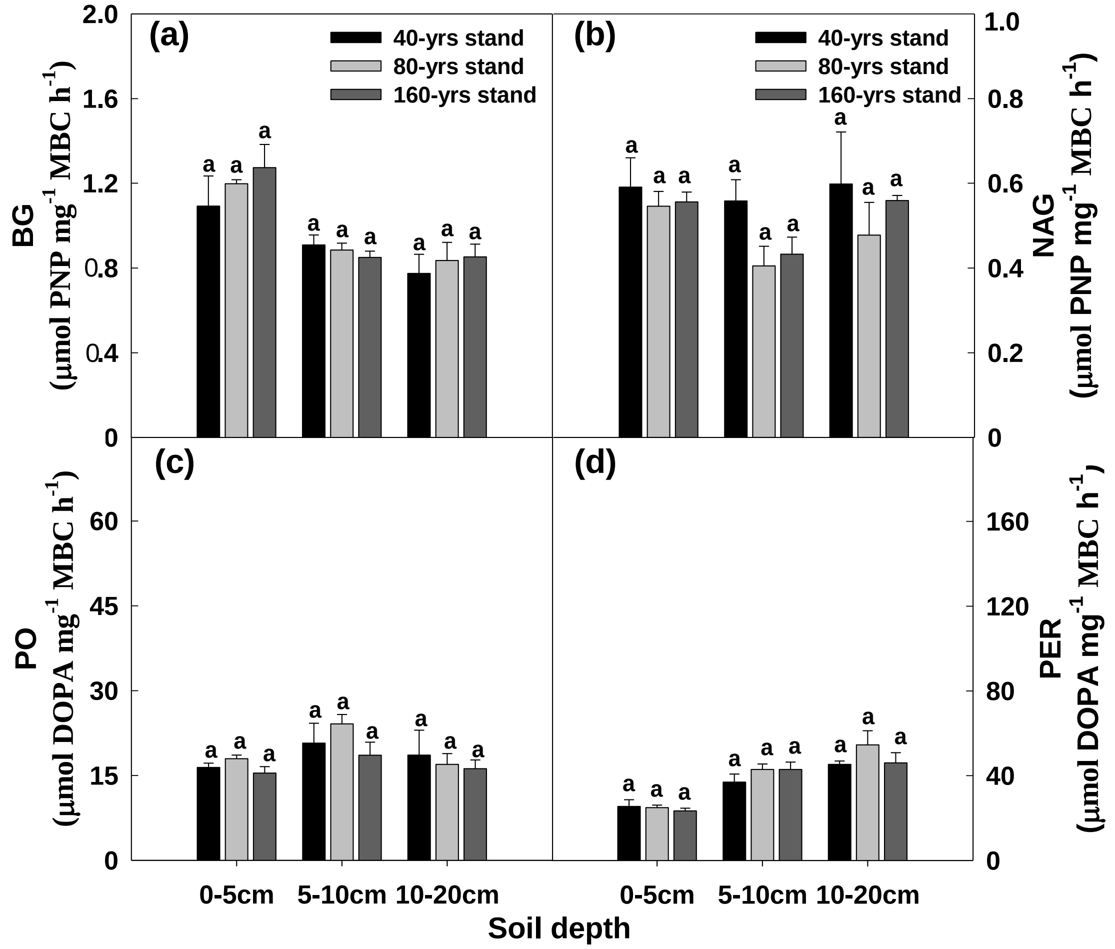

Supplement: Supplementary Figures [file srep22411-s2.pdf]
